# Is patient activation a mediator of the effect of a health promoting intervention in adults at high risk of type 2 diabetes? A longitudinal path model analysis within a randomised trial

**DOI:** 10.1186/s12889-022-12864-z

**Published:** 2022-03-04

**Authors:** Morten Frydenberg, Helle Terkildsen Maindal, Adam Fletcher, Lise Juul

**Affiliations:** 1 Department of Public Health, Aarhus University, Denmark, MFStat, Aarhus, Denmark; 2 Department of Public Health, Environments and Society, London School of Hygiene & Tropical Medicine, London, UK; 3 Department of Clinical Medicine, Danish Center for Mindfulness, Aarhus University, Aarhus, Denmark

## Abstract

**Background:**

Exploring mediators of preventive intervention effects has important implications for the planning of complex interventions. Our aim was to assess the extent to which knowledge, skills and confidence to manage health, here measured as “patient activation”, was a mediator of the effect of the intervention “Live your life without diabetes” on weight, waist circumference and systolic blood pressure at 12 months follow-up in adults at high risk of type 2 diabetes.

**Methods:**

Autoregressive path models with three time points of measurement, and contemporaneous and constant b paths were used in a randomised controlled trial (RCT). The RCT took place in a Danish municipal healthcare center and included 127 individuals aged 28 to 70 years with fasting plasma glucose: 6.1–6.9 mmol/l and/or glycated haemoglobin (HbA_1c_): 42.0–47.9 mmol/mol. Participants were randomised to routine care (*n* = 64), or intervention (*n* = 63). The intervention group received an empirical and theory-based intervention delivered over four two-h group sessions during five weeks, and two further sessions after one and six months. The outcomes were weight, waist circumference and systolic blood pressure, and the mediator was patient activation, measured by the self-reported Patient Activation Measure (PAM). Data for the present study was derived from questionnaires and clinical measures from baseline, three- and 12-months follow-up.

**Results:**

Mediated effects via PAM on: weight: − 0.09 kg (95% CI − 0.38 to 0.20) out of the total effect − 1.09 kg (95% CI − 3.05 to 0.87); waist circumference: − 0.04 cm (95% CI − 0.36 to 0.28) out of the total effect − 1.86 cm (95% CI − 4.10 to 0.39); and systolic blood pressure: − 0.31 mmHg (− 1.10 to 0.49) out of the total effect − 2.73 mmHg (95% CI − 6.34 to 0.87).

**Conclusion:**

We found no mediating effects of patient activation as a single variable of the intervention “Live your life without diabetes” on weight, waist circumference and systolic blood pressure at 12 months follow-up in adults at high risk of type 2 diabetes. Our study demonstrates an analytic approach for estimating mediating effects in complex interventions that comply with the criteria on temporal ordered data. Future studies should include possible interacting variables.

## Background

Physical activity and healthy diet are central factors for preventing several long-term diseases [1]. Efficacy trials have shown that goal-orientated interventions that focused on changes in diet, physical activity and weight in high-risk individuals of type 2 diabetes, cut the risk of progression to type 2 diabetes in half [2]. An efficacy trial showed that a weight loss > 5% at one-year was associated with prevention of type 2 diabetes at 3 years follow-up [3]. However, changing and sustaining behaviour is complex, and the pathways for which intervention effects occur remain unclear. It is a challenge for health care systems to identify appropriate, supportive intervention content that is effective under ‘usual circumstances’ and also reaches the intended target population.

Based on an efficacy trial [3] and health promotion theory, staff in a Danish municipal health care center developed a type 2 diabetes preventive intervention “Live your life without diabetes” [4]. They hypothesised that improved knowledge, skills and confidence to manage health among the participants would lead to behaviour change regarding diet and physical activity leading to weight loss as the primary 12-month outcome. The intervention “Live your life without diabetes” was evaluated in a randomised controlled trial (RCT) showing effects on waist circumference and systolic blood pressure; and pointed towards effect on weight, at 12 month among Danish adults at high risk of type 2 diabetes [4]. Furthermore, the trial pointed towards effect, however not statistically significant, on patient activation i.e., a person’s knowledge, skill and confidence related to managing health at three and 12 months. However, RCTs are designed to test whether interventions have the hypothesised effect on chosen outcomes, but they do not however, clarify how, for example whether patient activation led to the effects seen. Bonell et al. have suggested that mechanisms of change analyses are embedded within RCTs [5], and the recently revised Medical Research Council (MRC) framework for developing and evaluating complex interventions has also placed an emphasis on the importance of identifying mechanisms of change [6, 7]. A mechanism has been defined as “the process that is responsible for change”, and a mediator is a variable that account statistically for the association between an independent variable (e.g., intervention or not) and a dependent variable (an outcome measure e.g., weight) [8].

There is no ‘gold standard’ design for including tests of mechanisms of change within complex interventions in RCTs. However, it has been suggested that some criteria need to be fulfilled in order to test mechanisms or mediators of change of complex interventions: i) a clear association between change in the mediator and the outcome, and ii) that change in the mediator precedes change in the outcome [8]. The latter requires longitudinal study designs with mediators and outcome variables measured at, at least, three separated and ordered time points [8]. The hypothesised mechanisms between intervention activities, mediators and outcomes have been described as the intervention ‘programme theory’ [9, 10], which often are divided into a conceptual theory and an action theory [11]. The conceptual theory describes the association between the mediator and the outcome and the action theory describes, the degree to which the intervention affects the mediators [12]. The present study will particularly add new knowledge regarding the conceptural theory. Regarding the action theory, our previous analysis showed that the intervention had the following effect on PAM at 3 months: 3.0 (95% CI − 2.4 to 8.4) score points and; at 12 months: 3.2 (95% CI − 3.1 to 9.4) [4].

Structural equation modelling (SEM) has the ability of estimating multiple equations simultaneously, enabling analysis of mediated and unmediated effects simultaneously [13]. SEM may be a valuable method to combine with an RCT design for the investigation of proposed programme theories of complex interventions.

Therefore, we aimed to investigate the programme theory of the “Live your life without diabetes” intervention statistically; to assess i) the mediating effects via patient activation of the intervention on weight, waist circumference and systolic blood pressure at 12 months follow-up, and thereby also adding knowledge about the conceptual theory. Regarding the conceptual theory, we hypothesised that changes in PAM were associated with changes in weight, waist circumference and systolic blood pressure.

## Methods

### Design and setting of the study

**Fig. 1:**
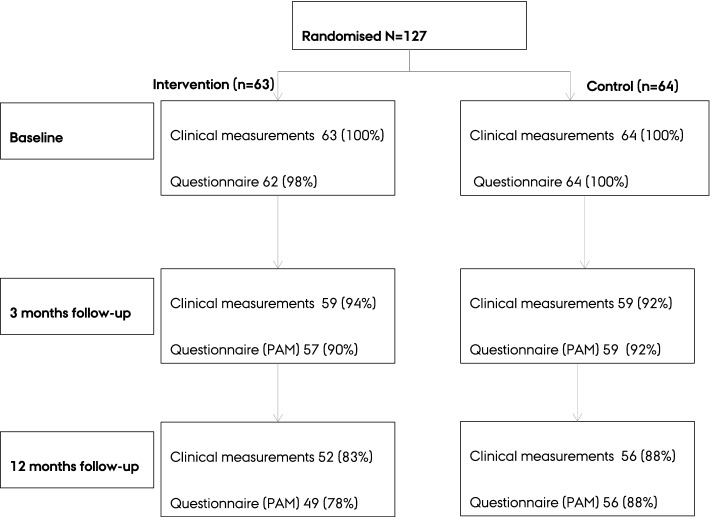
Trial profile. Clinical measurements refer to measurements of weight, waist circumference and blood pressure

In this study we used longitudinal path models of RCT data. This is a secondary, mediation analysis of the “Live your life without diabetes” trial, which has been reported and described in detail previously [4]. It was conducted among 127 individuals, who were referred from 16 general practices to the health care center in the municipality of Holstebro in Denmark. The inclusion criteria were; age < 70 years, and a measurement of fasting plasma glucose: 6.1–6.9 mmol/l (the thresholds for Impaired Fasting Glucose according to clinical guidelines) and/or glycated haemoglobin (HbA_1c_): 6.00–6.49% (42.0–47.9 mmol/mol) within the previous 6 months [4]. Participants were either randomised to a routine care group (*n* = 64) or the intervention group (*n* = 63) [4]. Table 1 shows participant characteristics and Fig. 1 shows the flowchart.

**Table 1:** Baseline characteristics of participants included in the “Live your life without diabetes” intervention trial in Holstebro Municipal Healthcare Center, Denmark 2011–13

Characteristics	Intervention (***n*** = 63)	Control (***n*** = 64)
*Demographic*		
Sex, men, n (%)	21 (33)	19 (30)
Age, median (quartile1, quartile3) (years)	58 (50, 63)	60 (51, 64)
Education, n (%)		
≤ 11 years	10 (16)	24 (38)
> 11 < 15 years	41 (66)	31 (49)
≥ 15 years	11 (18)	8 (13)
*Clinical*		
BMI, mean ± SD (kg/m^2^)	31.4 ± 5.5	30.4 ± 5.6
Weight, mean ± SD (kg)	88.9 ± 17.8	85.7 ± 16.0
Waist circumference, mean ± SD (cm)	106 ± 14	104 ± 11
Systolic blood pressure, mean ± SD (mmHg)	134 ± 16	132 ± 12
Diastolic blood pressure, mean ± SD (mmHg)	84 ± 9	81 ± 8
HbA1c, mean ± SD (mmol/mol)	40.7 ± 3.5	40.6 ± 3.9
Total cholesterol, mean ± SD (mmol/l)	5.1 ± 1.1	5.4 ± 1.0
LDL-cholesterol, mean ± SD (mmol/l)	3.1 ± 0.9	3.3 ± 0.9
HDL-cholesterol, mean ± SD (mmol/l)	1.3 ± 0.3	1.3 ± 0.3
*Self-reported patient activation*		
Patient Activation Measure, mean ± SD	62.8 ± 12.3	63.0 ± 14.8

*BMI* Body Mass Index, *HbA*_*1c*_ glycated haemoglobin, *HDL* high-density lipoprotein, *LDL* low-density lipoprotein, *SD* standard deviation

### Intervention

The intervention is described in detail previously [4]. It was delivered over four two-hour group sessions during five weeks, and two further sessions after one and six months. Health care staff in the Holstebro Municipal Health Care Center; a dietitian and an occupational therapist delivered the intervention. The pedagogical approach and the methods used were informed by Mezirow’s theory of transformative learning [14], health literacy theory [15] and dimensions of health knowledge and action competence [16].

### Measurements and outcomes

All data for the present study was derived from questionnaire data and clinical measures that were measured at baseline, three and 12 months after intervention.

In the present study, we used data on weight, waist circumference and systolic blood pressure as outcomes. These measurements were collected at the Holstebro Health Care Center by the same two trained health care staff following standard operating procedures with the same equipment throughout the study. Blood pressure was calculated as the mean of three measurements performed after at least 10 min rest, while participants were seated with the cuff on the right arm at the level of the heart. Height and weight were measured in light indoor clothing, without shoes. Waist circumference was recorded as the average of two measurements of waist circumference using a tape measure halfway between the lowest point of the rib cage and the anterior superior iliac crests when standing [4].

We assessed the mediator, patient activation, using the Patient Activation Measure (PAM) [17, 18]. PAM measures a person’s knowledge, skill and confidence related to managing health with items such as “I understand my health problems and what causes them”, “I know how to prevent problems with my health” and “I am confident I can figure out solutions when new problems arise with my health”. We used the short form 13-items PAM which had been translated and validated into Danish in a similar population [18]. Each item had five response categories: 1) disagree strongly, 2) disagree, 3) agree, 4) agree strongly, 0) not applicable/not able to answer. The PAM-activation scores and levels were calculated by Insignia Health [17, 19]. The activation score is based on a 0–100-point scale, with most individuals having activation scores between 30 and 90. Scores outside of this range are unlikely and generally result from individuals responding “Disagree Strongly” or “Agree Strongly” to each survey item [19]. Insignia Health states that each point change in activation score is meaningful, and a change of 3–4 points is associated with the difference between engaging and not engaging in particular behaviors [19]*.*

Age, sex and educational level were included as covariates. Education was categorised in three groups; 1) mandatory school and an optional 11th year of school at the most (≤11 years), 2) secondary education and/or vocational training< 3 years at the most (> 11 < 16 years) and, 3) secondary education and vocational training ≥3 years or tertiary education (≥16 years).

### Statistical analysis

In statistical mediation analysis, the conceptual theory; the association between the mediator and the outcome is termed the *b* path. The relationship between the intervention and the mediator is termed the *a* path. The total effect of the intervention on the outcome is termed *c*, and the effect of the intervention on the outcome adjusted for the mediator is termed *c´* [12].

**Fig. 2:**
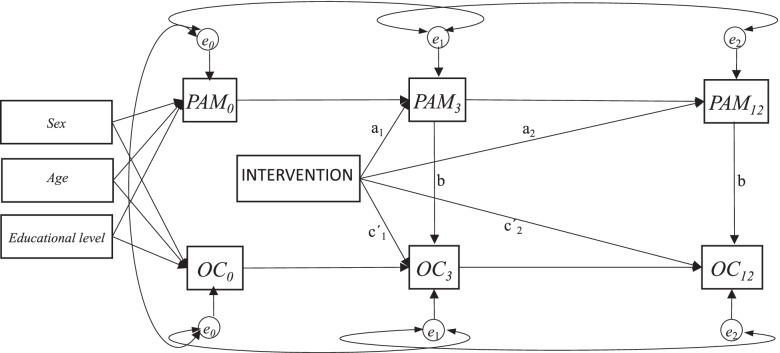
Autoregressive mediation model showing the *a*, *b* and *c´* paths. The *a* paths represent the association between the intervention and PAM (the action theory); The *b* paths represent the association between PAM and the outcomes (the conceptual theory); All paths including ≥1 PAM measure contribute to the calculation of the mediated effects. The *c´* paths represents the intervention effect on the outcomes adjusted for PAM. All paths from intervention to outcomes not via PAM contribute to the calculation of the unmediated effects. At baseline residual covariance between all mediators and outcome. For each mediator and the outcome residual covariance over time. PAM: Patient Activation Measure; OC: Outcome

We analysed the data using autoregressive models, with three time points of measurement, and contemporaneous and constant *b* paths [12, 20] (see Fig. 2). We fitted models in the SEM framework in Stata 16, using full information maximum likelihood and conditioning on covariates to account for missing data under the missing at random assumption.

In all models we adjusted baseline values of mediators and outcome for the following covariates: sex, age and educational level. We allowed for correlation between measurement errors for: mediator and outcome at baseline, two consecutive measurements of the mediator; and two consecutive measurements of the outcome, as illustrated in Fig. 2.

In order to estimate the mediated effect of the intervention on outcomes at 12 months in the models, we first identified all paths that went from intervention to outcome at 12 months through any measure of the mediator, which was in total three paths e.g., Intervention- > PAM_3_- > PAM_12_- > OC_12_. We multiplied the coefficients within each of these three paths and added the path specific products to obtain the mediated effect. The unmediated effect was estimated based on paths from intervention to outcome at 12 months not passing the mediator (all paths from intervention going to outcomes at 12 months that started with a direct path from intervention to outcome); two paths in total e.g., Intervention- > OC_3_- > OC_12_. The total effect was the combined effect via all paths from intervention to outcome (the sum of all the mediated effects and the unmediated effect).

We calculated the 95% Confidence Intervals (CI) of the estimates of the overall *a* paths, the mediated, the unmediated and the total effect, by use of 50 bootstrap replications. We multiplied the *b* paths estimates with 10 in order to present associations of a 10-point increase in PAM with expected changes in the outcomes.

## Results

Our study population had a median age of 59 years, range 28–70 years. A total of 87 (68.5%) were women, and a total of 34 (27%) had less than 11 years of education. The mean baseline PAM score was 62.9 (SD 13.6), and the mean clinical measures at baseline were as follows; Body Mass Index (BMI): 30.9 kg/m^2^ (SD 5.6); weight: 87.3 kg (SD 16.9); waist circumference: 105.0 cm (SD 12.6); Systolic blood pressure: 133.1 mmHg (SD 14.1); diastolic blood pressure: 82.3 (SD 8.8); HbA1_c_: 40.6 mmol/mol (SD 13.9); Total cholesterol: 5.2 mmol/l (SD 1.1), and low-density lipoprotein (LDL)-cholesterol: 3.2 mmol/l (SD 0.9).

The results of our models showed no statistically significant mediated, unmediated or total effects for any of the outcomes (Table 2). Results of the *a* path estimates in the model with weight as outcome suggested that the intervention increased the PAM by 3.36 score points (95% CI − 1.62 to 8.34) at 3 months; and in total by 3.96 score points (95% CI − 2.30 to 10.22) at 12 months (Table 2). Similar results for the *a* paths were found in the models with waist circumference and systolic blood pressure as outcomes. Regarding the *b* path estimates, our results suggested that every 10 score-point increase in PAM were associated with an expected weight loss of 0.13 kg i.e., 130 g (95% CI − 100 to 360); an expected waist circumference reduction of 0.05 cm (95% CI − 0.30 to 0.41); and an expected decrease in systolic blood pressure of 0.43 mmHg (95% CI − 0.19 to 1.05) (Table 2). Therefore, our results showed no further indications of mediated effects via PAM on weight, waist circumference or systolic blood pressure at 12 months (Table 2). The overall mediated effect via PAM on weight was − 0.09 kg (95% CI − 0.38 to 0.20) out of the total effect − 1.09 kg (95% CI − 3.05 to 0.87); on waist circumference: − 0.04 cm (95% CI − 0.36 to 0.28) out of the total effect − 1.86 cm (95% CI − 4.10 to 0.39); and on systolic blood pressure: − 0.31 mmHg (95% CI − 1.10 to 0.49) out of the total effect − 2.73 mmHg (95% CI − 6.34 to 0.87).

**Table 2:** Mediated and unmediated effects via patient activation measure (PAM) for the “Live your life without diabetes” intervention on weight, waist circumference and systolic blood pressure at 12 months follow-up in adults at high risk of type 2 diabetes (*n* = 127)^a^

Outcome (OC)		Weight (kg)	Waist circumference (cm)	Systolic blood pressure (mmHg)
		estimate (95% CI)^b^	estimate (95% CI)^b^	estimate (95% CI)^b^
**Path coefficients**				
*a* paths				
*a*^1^	Intervention- > PAM_3_	3.36 (−1.62 to 8.34)	3.78 (−1.21 to 8.76)	3.42 (− 1.55 to 8.38)
*a*^2^	Intervention- > PAM_12_	0.13 (−6.21 to 6.47)	2.08 (−3.20 to 7.36)	0.27 (−5.94 to 6.48)
overall *a*	Intervention- > − > PAM_12_	3.96 (−2.30 to 10.22)	4.26 (− 1.52 to 10.04)	4.05 (− 2.08 to 10.17)
*b* paths (constrained)	PAM (per 10 score points) - > OC	−0.13 (−0.36 to 0.10)	−0.05 (−0.41 to 0.30)	−0.43 (− 1.05 to 0.19)
**Effects**				
Mediated	Intervention- > PAM- > OC_12_ (all paths including ≥1 PAM measure)	−0.09 (− 0.38 to 0.20)	−0.04 (− 0.36 to 0.28)	−0.31 (− 1.10 to 0.49)
Unmediated	Intervention- > OC_12_ (all paths not via PAM)	−1.00 (− 3.05 to 1.05)	− 1.82 (−4.12 to 0.49)	−2.42 (− 6.18 to 1.34)
Total (*c*)		−1.09 (− 3.05 to 0.87)	−1.86 (− 4.10 to 0.39)	−2.73 (− 6.34 to 0.87)
Successful bootstrap out of 50		45	50	46

At baseline residual covariance between all mediators and outcome. At each follow-up time residual covariance between all mediators. For each mediator and the outcome residual covariance over time

^a^ According to Fig. 1. ^b^Adjusted for sex, age, educational level

## Discussion

This study demonstrates an analytic approach for estimating mediating effects in complex intervention research. At baseline our study population had a mean PAM at 62.9 corresponding to a mean level that indicates an incipient engagement in recommended health behaviours, but not proactive behaviour and engagement in many health behaviour recommendations [21]. Hence, there was a room for improvement in PAM at baseline, and there were also room for improvement in the clinical outcomes.

We found no statistically significant effects via patient activation using the PAM as a mediating factor. However, like our previous results [4], our *a* path estimates pointed in the direction, indicating support for the action theory, i.e., that the intervention increased PAM (a person’s knowledge, skill and confidence related to managing health) in adults at high risk of type 2 diabetes. However, the result was not statistically significant and was associated with an uncertainty that the intervention could decrease PAM by 2 score points or increase PAM by 10 score points. Our results also pointed in the direction of potential effects on weight, waist circumference and systolic blood pressure at 12 months, however, again these results were associated with uncertainty and not statistically significant. Taken together, these results indicate a lack of statistical power. On the other hand, the *b* path estimates showed no or minor association between PAM and weight, waist circumference and systolic blood pressure. Our confidence intervals showed no relevant change in the outcomes associated with a 10-score point change in PAM. The highest expected weight loss associated with a 10-point increase in PAM would be 360 g, the highest expected reduction in waist circumference associated with a 10-point increase in PAM would be 0.41 cm and the highest expected decrease in systolic blood pressure associated with a 10-point increase in PAM would be 1.5 mmHg (Table 2). Therefore, even though our study may have lacked statistical power, our confidence intervals show that even in the “very best” scenarios the impact of PAM on the outcomes would be quite low. A clear association between change in the mediator and the outcome i.e., a valid conceptual theory is an obvious requirement for mediation [8].

We found no indications of mediating effects via PAM for the intervention “Live your life without diabetes” on weight, waist circumference and systolic blood pressure at 12 months follow-up.

Miller et al. found that PAM increased 9.7 points in people with type 2 diabetes participating in the DESMOND intervention [22]. They assumed a more than five points increase in PAM to be a clinically significant change. However, they did not analyse the association between the changes in PAM with clinical change. Hibbard et al. has shown that positive change in PAM was associated with positive change in health behaviour [23]. Harvey et al., on the other hand, found no relation of changes in PAM and BMI [24]. Hosseinzadeh H, et al. assumed in their review that patient activation would be a major driver of type 2 diabetes self-management interventions if RCTs showed effect on both PAM and clinical outcomes [25]. Our RCT was included in their review. However, our current mediation analysis based on this RCT showed no indications of PAM as a mediator for our intervention in people at high risk of type 2 diabetes on clinical outcomes at 12 months. This highlights the importance of adding mediation analysis in complex intervention research.

Our study population was recruited as a high-risk population for developing type 2 diabetes. Research had shown that a weight loss > 5% from the initial weight could have a major preventive impact [3]. Our baseline data showed potential for weight loss and also indicated a cardiovascular risk profile in terms of high waist circumference and cholesterol level. A recent study showed that lack of awareness of cardiovascular risk in terms of hypertension and hypercholesterolemia is relatively frequent in the general population and is associated with significantly higher arterial stiffness [26]. The “Live your life without diabetes” intervention aimed at improving the participants´ knowledge, skills and confidence to manage health. However, it did not seem to be sufficient in order to change the outcomes adequately within 12 months. Systematic development of complex interventions is very important and often require knowledge from many studies with diverse designs and methods [6, 7]. Our study contributes with such knowledge as it may help to improve the understanding of, which mechanisms that may lead to the intended change, thereby providing input to the design of future programme theories. Knowledge that may help inform programme theory will help inform intervention developers and further the transferability of the active intervention components across settings [6, 7]. A recent review found that it is common to build health behavior interventions in type 2 diabetes care on theoretic models [27]. One recent example is the English National Health Service Diabetes Prevention Programme aiming to prevent the progression to Type 2 diabetes in high-risk adults, through changing diet and physical activity behaviors [28]. Hawkes et al. published a post hoc proposal of a programme theory based on a variety of behaviour change theories, that outlined how different concepts (e.g., self-efficacy) were expected to relate to the intended primary outcomes [28]. It is important that such proposals of programme theories are thoroughly empirically investigated and tested in order to provide useful knowledge [6, 7]. The new MRC framework emphasizes the importance of building complex interventions on the existing evidence and an explicit theoretical basis. It also emphasizes involving all relevant stakeholders in the early phases of intervention development [6, 7]. Therefore, future research should also involve representatives from the target group at high risk of type 2 diabetes in the early phases of development of programme theories, before testing them.

### Strengths and limitations

To our knowledge, this is the first study to investigate PAM as a mediator of the effect of a complex health promoting intervention on health outcomes by use of longitudinal mediation models in an RCT. We established a timeline with three time points of measurements, and used a theory as an underlying guide. Furthermore, we analysed the mediated and unmediated effects simultaneous by use of longitudinal path models inspired by Goldsmith et al. [20]. Attrition rate in the trial was low, and no characteristics were found to be statistically significant associated with attrition [4]. Furthermore, in our analysis, we took into account for missing data.

We assumed the relationship between mediator and outcome (conceptual theory) to be reasonably consistent over time. Therefore, we made the *b* paths equal as constant *b* paths should provide more precise estimation [20]. We choose to use contemporaneous *b* paths [20], because we expected that changes in the PAM, as well as the outcomes, began during the intervention, occurring before the first post randomisation measurement [20]. Our results of the *a* paths (Table 2) and previously published total effects also showed that the intervention effects, however not statistically significant, probably occurred already at the first post measurement point at 3 months [4]. We complied with the criteria on temporal ordered data by having three timepoints of measurement. However, one could argue that three measurement points are too sparse. Including more measurement points results in better data for investigating mediating effects of interventions.

Maindal et al. had previously validated the Danish version of PAM in a study population similar to the present [18]. They concluded that PAM was an acceptable valid and reliable measurement to measure patient activation in the Danish context. Unfortunately, in our longitudinal models, it was not feasible to adjust for measurement error of latent variables as suggested by Goldsmith et al. [20]. As they only illustrated the application of the model on simulated data, their tutorials were not compatible with real data [20]. Hence, it is a limitation that our models only include PAM scores and not the measurement part of the PAM scores i.e., the item responses. Hence, our models include measurement error.

It is also a limitation that our analysis only included one possible mediator variable, and not e.g., health literacy or action competence. The effects may have been caused by the interaction of these variables, and future mediation models should include all the most evident mediating variables based on the programme theory. This was done in a recent study evaluating mediators for the effects of a complex intervention, however in another context [29].

According to the confidence intervals, the statistical power may be a limitation of our study with regard to the assessment of the action theory and the total effects of the intervention. However, regarding the assessment of the conceptual theories and the mediated effects, the confidence intervals indicated no relevant impact of PAM on the clinical outcomes.

## Conclusions

We found no support for mediating effects of patient activation for the intervention “Live your life without diabetes” on weight, waist circumference and systolic blood pressure at 12 months follow-up in adults at high risk of type 2 diabetes. Our study demonstrates an analytic approach for estimating mediating effects in complex intervention research that comply with the criteria on temporal ordered data (e.g., at three measurement points).

## Data Availability

The dataset used and analysed during this study is available from the corresponding author by reasonable request.

## Abbreviations

BMI: Body Mass Index
CI: Confidence Intervals
HbA_1c_: Glycated haemoglobin
HDL: High-density lipoprotein
LDL: Low-density lipoprotein
MRC: Medical Research Council
OC: Outcome
PAM: Patient Activation Measure
RCT: Randomised controlled trial
SEM: Structural equation modelling
SD: Standard deviation
